# Supplementary material for: Uncertainty about social interactions leads to the evolution of social heuristics
Source: Nat Commun. 2018 May 31;9:2151. doi: 10.1038/s41467-018-04493-1 (PMC5981325; doi:10.1038/s41467-018-04493-1)
Supplement: Supplementary file 1 — Supplementary Information [file 41467_2018_4493_MOESM1_ESM.pdf]

Supplementary Information

Uncertainty about social interactions leads to the evolution of social heuristics

van den Berg & Wenseleers

CONTENTS

Supplementary Table 1 2

Supplementary Note 1: Invasion analysis 3

Supplementary Figure 1 3

Supplementary Note 2: Simulations for fixed values of c 4

Supplementary Figure 2 4

Supplementary Figure 3 5

strategy	*name*	*description*
0 0 0 0	*ALLD*	always defects
0 0 0 1	*desperate*	only cooperates after mutual defection
0 0 1 0	*Acon-D*	anti-conventional, shifts after playing opposite of opponent, otherwise defects
0 0 1 1	*inconsistent*	plays opposite of previous move
0 1 0 0	*con-D*	conventional, stays after playing the opposite of opponent, otherwise defects
0 1 0 1	*ATFT*	anti-tit for tat, plays opposite of opponent's last move
0 1 1 0	*APavlov*	win, shift; lose, stay
0 1 1 1	*hopeless*	only defects after mutual cooperation
1 0 0 0	*grim*	only cooperates after mutual cooperation
1 0 0 1	*Pavlov*	win, stay; lose, shift
1 0 1 0	*TFT*	tit for tat, copies opponent's last move
1 0 1 1	*MNG*	Mr Nice Guy, only defects after 'being cheated' (playing C while other plays D)
1 1 0 0	*consistent*	repeats its own previous move
1 1 0 1	*con-C*	conventional, stays after playing the opposite of opponent, otherwise cooperates
1 1 1 0	*willing*	only defects after mutual defection
1 1 1 1	*ALLC*	always cooperates

Supplementary Table 1. An overview of all 16 possible substrategies that condition their behaviour on the previous interaction round. The first column (strategy) shows whether the strategy defects (0) or cooperates (1) after each of the four possible outcomes of the previous round (from left to right: mutual cooperation, having cooperated while the interaction partner defected, having defected while the interaction partner cooperated, and mutual defection).

Supplementary Note 1: Invasion analysis

Supplementary Figure 1 shows the invasion fitness of the most common heuristic (*grim*) and context-dependent strategies (*grim*/*pavlov* – see Fig. 3 in the main text) against each other, for different values of uncertainty. The most common heuristic strategy can invade the most common context-dependent strategy only under high uncertainty (if $u \geq 0.6$), whereas the most common context-dependent strategy can never invade the most common heuristic strategy. For most of the range, neither strategy can invade the other. Nonetheless, as might be expected, the invasion fitness of the heuristic strategy increases with uncertainty, whereas the invasion fitness of the context-dependent strategy decreases with uncertainty. Hence, in finite populations (like in our model), the probability of the heuristic strategy invading a population of the context-dependent strategy becomes more likely with increasing uncertainty, whereas opposite is true for invasions of the context-dependent strategy. Of course, the invasion fitnesses of these two strategies against each other only tell a small part of the story – they only comprise a tiny part of the strategy space in our model.

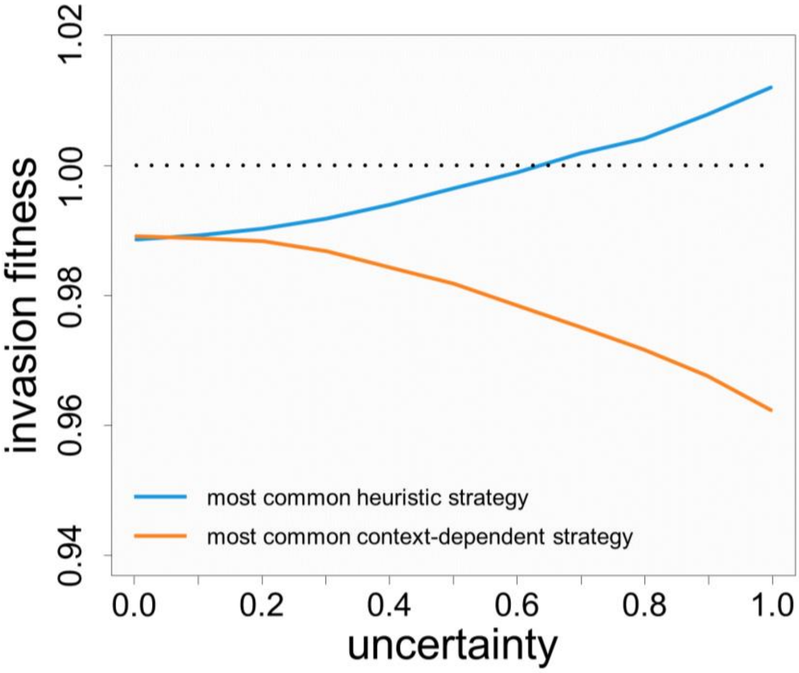

Supplementary Figure 1. Invasion fitness of the most common heuristic strategy (*grim* with probability to cooperate on the first move of 0.99) and the most common context-dependent strategy (substrategy 1: *grim* with probability to cooperate on the first move of 0.30; substrategy 2: *pavlov* with probability to cooperate on the first move of 0.83; threshold between both strategies: c = -0.46) against each other. If the invasion fitness exceeds 1.00 (dotted line), the strategy can invade the other. To obtain these values, we simulated 100,000 interactions of both strategies against each other and against themselves, for each level of uncertainty (from 0 to 1 in steps of 0.1).

Supplementary Note 2: Simulations for fixed values of *c*

Supplementary Figure 2 shows the cooperation rate at the end of simulations in which all interactions were characterized by a fixed consequence of cooperation (c). The mean cooperation level over the entire range of c equals 0.55 (based on an interpolation of data points in the region between -3.0 and -2.0 and the region between -0.5 and 1.0 when assuming linearity in these regions). This exceeds the cooperation level that we observe in the original model under low uncertainty (0.46) but is far below the observed cooperation level in the original model under high uncertainty (0.87).

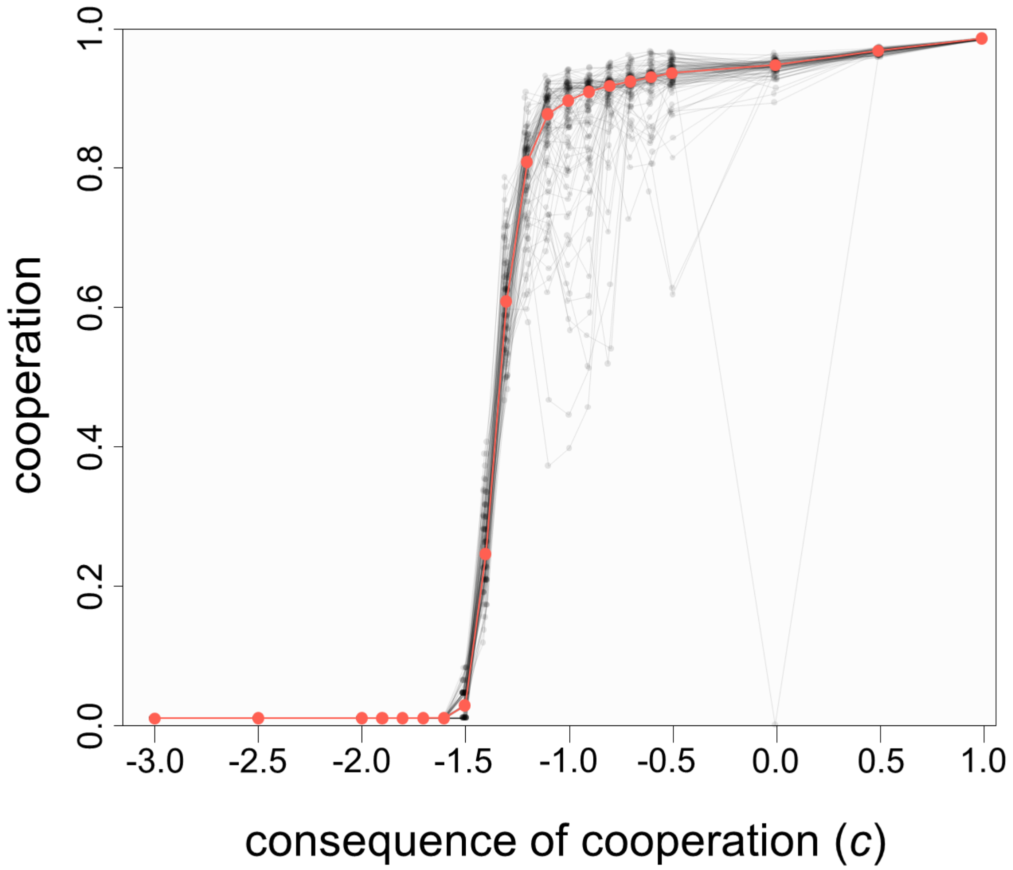

Supplementary Figure 2. Cooperation levels at the end of the simulations of the model without heterogeneity in interaction types, for various values of c. Each grey dot represents the cooperation level in the last generation of the simulation for a single mutation matrix (see main text), averaged over 50 replicate simulations. Simulations for 100 different mutation matrices are shown. The red line/dots represent the median of the average cooperation levels of the different mutation matrices.

The average cooperation levels that we observe under high uncertainty in our original model (0.87) are similar to the average cooperation levels that we observe for the model without heterogeneity at $c = -1$ (0.85). Since $c = -1$ is the expected consequence of cooperation in our original model, this may lead to the suspicion that individuals are selected to play 'the average game' when uncertainty is maximal. To investigate this, Supplementary Figure 3 shows an overview of the strategies that evolved in both situations. Although the simulation outcomes in the original model under full uncertainty and in the model with fixed $c = -1$ are not entirely the same, they are very similar. In both

cases, the evolution of the strategy *grim* was by far the most likely outcome of the simulations, and the strategy *tit for tat* was also relatively likely to evolve. However, there are also some differences. In the model with fixed *c*, the strategies *desperate, alld* and *pavlov* were more likely to evolve than in the original model. In contrast, in the original model some simulations led to the evolution of context-dependent strategies (even though the information on which these strategies condition their behaviour is completely unreliable). Also, simulation outcomes where no single strategy dominated the population (*i.e.*, had a frequency of >0.8) at the end of the simulations occurred relatively frequently in the original model, whereas they never occurred in the model with fixed *c*.

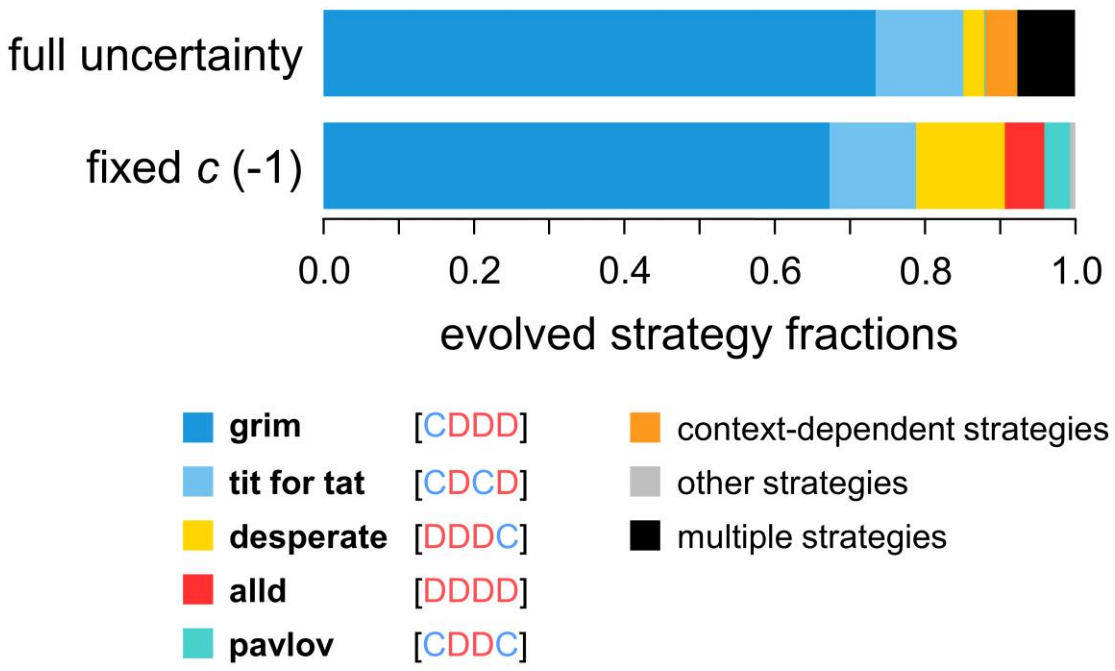

Supplementary Figure 3. Evolved strategies in the original model under full uncertainty (upper bar) and the model without heterogeneity, for $c = -1$ (lower bar). Each bar shows the fraction of simulations that led to the evolution of each of the mentioned strategies dominating the population (*i.e.*, reach a frequency > 0.8). Both bars represent a total of 5,000 simulations (50 replicate simulations for each of 100 mutation matrices.
